# Rapid Morphological and Cytoskeletal Response to Microgravity in Human Primary Macrophages

**DOI:** 10.3390/ijms20102402

**Published:** 2019-05-15

**Authors:** Cora Sandra Thiel, Svantje Tauber, Beatrice Lauber, Jennifer Polzer, Christian Seebacher, Rainer Uhl, Srujana Neelam, Ye Zhang, Howard Levine, Oliver Ullrich

**Affiliations:** 1 Institute of Anatomy, Faculty of Medicine, University of Zurich, Winterthurerstrasse 190, 8057 Zurich, Switzerland; svantje.tauber@uzh.ch (S.T.); beatrice.lauber@anatomy.uzh.ch (B.L.); jennifer.polzer@uzh.ch (J.P.); 2 Department of Machine Design, Engineering Design and Product Development, Institute of Mechanical Engineering, Otto-von-Guericke-University Magdeburg, Universitätsplatz 2, 39106 Magdeburg, Germany; 3 TILL I.D. GmbH, Am Klopferspitz 19a, 82152 Martinsried, Germany; seebacher@till-id.com (C.S.); rainer_uhl@me.com (R.U.); 4 National Aeronautics and Space Administration (NASA), ISS Utilization and Life Sciences Division, Kennedy Space Center, Cape Canaveral, FL 32899, USA; neelamsrjn@gmail.com (S.N.); ye.zhang-1@nasa.gov (Y.Z.); howard.g.levine@nasa.gov (H.L.); 5 Ernst-Abbe-Hochschule (EAH) Jena, Department of Industrial Engineering, Carl-Zeiss-Promenade 2, 07745 Jena, Germany; 6 Zurich Center for Integrative Human Physiology (ZIHP), University of Zurich, Winterthurerstrasse 190, 8057 Zurich, Switzerland; 7 Space Life Sciences Laboratory (SLSL), Kennedy Space Center, 505 Odyssey Way, Exploration Park, FL 32953, USA

**Keywords:** live cell imaging, suborbital rocket, microgravity, immune cells, cytoskeleton, nucleus

## Abstract

The FLUMIAS (Fluorescence-Microscopic Analyses System for Life-Cell-Imaging in Space) confocal laser spinning disk fluorescence microscope represents a new imaging capability for live cell imaging experiments on suborbital ballistic rocket missions. During the second pioneer mission of this microscope system on the TEXUS-54 suborbital rocket flight, we developed and performed a live imaging experiment with primary human macrophages. We simultaneously imaged four different cellular structures (nucleus, cytoplasm, lysosomes, actin cytoskeleton) by using four different live cell dyes (Nuclear Violet, Calcein, LysoBrite, SiR-actin) and laser wavelengths (405, 488, 561, and 642 nm), and investigated the cellular morphology in microgravity (10^−4^ to 10^−5^ g) over a period of about six minutes compared to 1 g controls. For live imaging of the cytoskeleton during spaceflight, we combined confocal laser microscopy with the SiR-actin probe, a fluorogenic silicon-rhodamine (SiR) conjugated jasplakinolide probe that binds to F-actin and displays minimal toxicity. We determined changes in 3D cell volume and surface, nuclear volume and in the actin cytoskeleton, which responded rapidly to the microgravity environment with a significant reduction of SiR-actin fluorescence after 4–19 s microgravity, and adapted subsequently until 126–151 s microgravity. We conclude that microgravity induces geometric cellular changes and rapid response and adaptation of the potential gravity-transducing cytoskeleton in primary human macrophages.

## 1. Introduction

The monocyte-macrophage system (MMS) belongs to the first line of immune defense, acts as a crucial effector system for attacking and killing bacteria and routinely clears more than one billion apoptotic cells from almost all tissues of the organism [1,2], thereby maintaining homeostasis of the human immune and tissue systems. Previous studies have reported disturbed cytokine release [3,4], significant changes in gene expression associated with macrophageal differentiation [5], signal transduction changes [6,7] and alteration of the cytoskeleton [8] in cells of the MMS in microgravity. Cytoskeletal alterations have been found in several tumor cell types after minutes [9,10,11] and after hours [12,13] in microgravity, whereby no changes of the actin network were detected in primary cardiomyocytes after four days microgravity [14]. Tumor cells are known to differ from non-malignant cells in terms of cytoskeletal organization, mechanics and regulation [15,16,17,18,19,20], loss of actin filaments [21] and disorganization of microtubules [22].

During the CELLBOX-PRIME International Space Station (ISS) experiment, we recently demonstrated, that 11 days in microgravity resulted neither in quantitative nor structural changes of the actin and vimentin cytoskeleton in primary human macrophages when compared to 1 g controls [23]. On the other side of the time scale, we recently found that the oxidative burst reaction rapidly responds to microgravity within less than one second [24,25] and fully adapted in less than one minute [25]. Parabolic flight and suborbital ballistic rocket experiments revealed a rapid coupling between gravitational forces and the transcriptome in human myelomonocytic U937 cells, occurring in the time frame of fewer than 20 s [26]. Our findings confirmed the discovery of rapid reactions to microgravity in macrophages more than 20 years ago by Armstrong et al. [27].

Thus, the questions arise if the stable cytoskeletal phenotype in human primary macrophages represents a stable “steady state” after adaptation to the new microgravity environment, and when these putative adaptive processes are occurring. Due to the importance of the macrophage system for the immune system and tissue homeostasis, response and adaptation mechanisms to altered gravity are crucial areas of concern for human health and performance during long-term space missions.

Whereby several types of cultured cells are sensitive to gravity [28,29], the immune system belongs to the most affected systems during spaceflight as reviewed by references [30,31,32]. The sensitivity of cells of the human immune system to reduced gravity has been confirmed in numerous studies in real and simulated microgravity [32,33]. However, with the exception of a very few studies [25], data are usually obtained as end-point-measurements, allowing no or only very limited information about dynamic processes, whereby investigation of cellular adaptation in altered gravity environments requires dynamic measurements and live imaging technologies. It seems also not reasonable to focus on any single mechanism in isolation [34,35], but respecting the reality that multiple simultaneous signals are transduced and integrated within the structural complexity of the living cell [28]. In this context, a live microscopy is an excellent tool for the characterization of cellular structures and small organisms, and in the last 30 years the technology evolved enormously. Nowadays, based on the advanced technology of confocal microscope systems, high-resolution structures can be recorded in fixed, as well as in living organisms [36,37,38]. Contrary to the current microscopy methods in standard laboratories, the implementation of live cell high resolution imaging in space flight experiments is not yet equally advanced. Until recently, only relatively simple optical systems existed for life sciences, which consisted of a simple camera to record smaller organisms, such as cichlid fish or daphnia [39,40], light microscopy to record bright field images, or epifluorescence systems for fluorescence-labelled cells [41,42,43,44]. These systems did not allow high-resolution imaging of subcellular structures in living cells. The development and construction of the FLUMIAS (Fluorescence-Microscopic Analyses System for Life-Cell-Imaging in Space) confocal laser spinning disk fluorescence microscope allows high-resolution images of living cells and subcellular structures on parabolic flights and suborbital rocket flights. A recent live imaging study with the FLUMIAS microscope reported disturbances of actin bundles in the cytoplasm of human thyroid carcinoma cells immediately after the onset of microgravity in a parabolic flight and suborbital ballistic rocket experiments, using Lifeact-GFP transfection [11], but without providing quantitative data. Because Lifeact-GFP induces severe artefacts at the cellular level, including F-actin organization, cellular morphology and biophysical behavior [45], we have instead chosen SiR-actin stain for live-cell imaging of the cytoskeleton [46], a newly developed fluorescent probe, which does not require transfection and peptide over expression. Since cytoskeletal organization and regulation in tumor cells is very different from non-malignant cells [15,16,17,18,19,20,21,22] and to some extent similar to the “microgravity” phenotype [23] described in previous studies [11], we used human primary macrophages [23] as test system instead of tumor cells.

In the present study we performed a live imaging experiment using the FLUMIAS confocal laser spinning disk fluorescence microscope during the TEXUS (German: Technologische Experimente unter Schwerelosigkeit)-54 suborbital ballistic rocket mission. We simultaneously labeled four different cellular structures (nucleus, cytoplasm, lysosomes, actin cytoskeleton) in primary human macrophages using four different live cell dyes and laser wavelengths of 405, 488, 561, and 642 nm, and investigated the cellular morphology in high quality microgravity (10^−4^ to10^−5^ g; Figure S1) over a period of about six minutes. We were able to observe for the first time, and live in primary human macrophages, rapid changes in the volume and surface of cell nuclei and cells in response to microgravity and rapid response and adaptation of the actin-cytoskeleton.

## 2. Results

**Figure 1:**
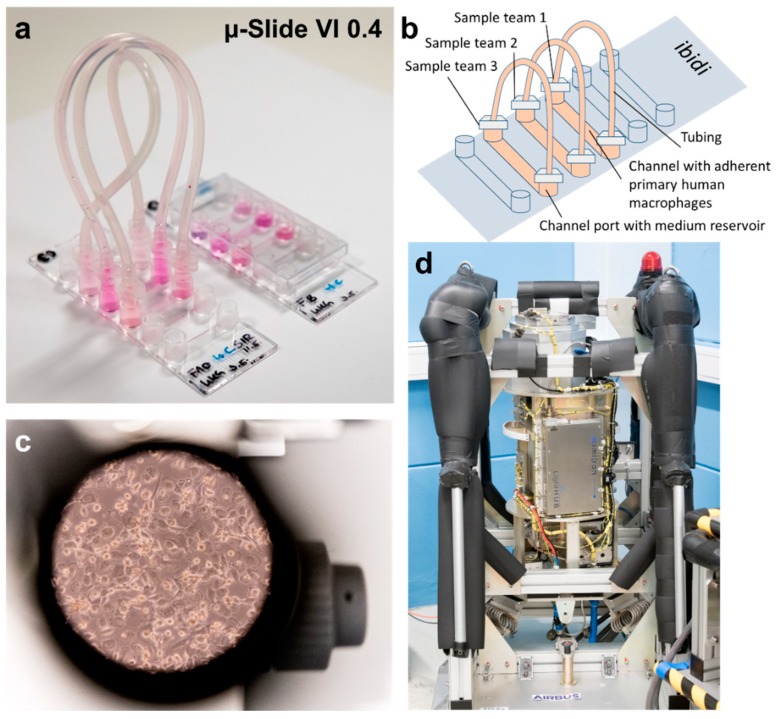
FLUMIAS TEXUS-54 experiment hardware. (**a**) An ibidi ibiTreat μ-Slide 0.4 (ibidi µ-Slide) prepared for flight; (**b**) Schematic of flight configuration of an ibidi µ-Slide closed with luer plugs and tubings. Three science teams seeded different cell types into three respective channels; (**c**) Primary human macrophages visualized by light microscopy. (**d**) The confocal laser spinning disk fluorescence microscope FLUMIAS, engineering model.

The confocal laser spinning disk fluorescence microscope FLUMIAS (Fluorescence-Microscopic Analyses System for Life-Cell-Imaging in Space, abbreviated as FLUMIAS microscope) (Figure 1), developed and built by Airbus DS for research during parabolic and suborbital ballistic rocket flights, provides the unique opportunity of observing living cells in microgravity for up to six minutes. Four different fluorescence colors can be used, since the microscope is equipped with four lasers with the wavelengths 405, 488, 561, and 642 nm.

During the TEXUS-54 campaign, we were able to demonstrate the full capacity of the FLUMIAS microscope with all four wavelengths in parallel. With the live cell dyes Nuclear Violet, Calcein, LysoBrite, and SiR-actin, which were excited by lasers of 405, 488, 561, and 642 nm wavelengths respectively, we visualized nuclei, cytoplasm, lysosomes, and the actin cytoskeleton in primary human macrophages simultaneously (Table 1 and Table 2).

The primary human macrophages were seeded into a channel of an ibidi ibiTreat μ-Slide 0.4 (ibidi µ-Slide) (Figure 1a–c). In total, three different teams participated in the FLUMIAS TEXUS-54 experiment (Figure 1). Each team worked with different cells and cellular markers. Due to the fact that three teams participated in the same experiment to demonstrate the capability of the instrument and to obtain a first insight into the effect of microgravity on different cell types, measurement time for each team was restricted: The measurements of one group, lasting between 21 and 22 s were interrupted by the measurements of the two other groups, leading to a break in recording of around 50 s.

Differentiated human primary macrophages were stained 24 h with SiR-actin and with Nuclear Violet Calcein, LysoBrite and SiR-actin 5.5 h before lift-off. Subsequently the three teams evaluated their respective channels on several prepared ibidi µ-Slides, the best one was selected for flight, mounted into the late access unit of the FLUMIAS microscope and inserted into the temperature-controlled microscope body in the payload.

**Figure 2:**
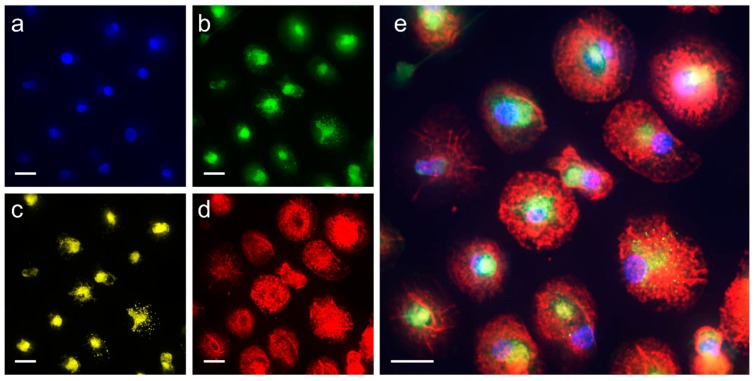
Live cell microscopy of primary human macrophages in microgravity with the confocal laser spinning disk fluorescence microscope FLUMIAS. Primary human macrophages stained with the live cell dyes: (**a**) Nuclear Violet for nuclei; (**b**) Calcein for the cytoplasm; (**c**) LysoBrite for lysosomes; and (**d**) SiR-actin for F-actin; were imaged in microgravity at four consecutive time points (t1_F: T+117–142 s, t2_F: T+192–217 s, t3_F: T+267–292 s, t4_F: T+342–367 s). Displayed is the measurement of t1_F; (**e**) overlay of all colors. Scale bars: 20 µm.

Then, an overview was generated and three positions selected, one main measurement position and two reserve positions. 10 min before the lift-off, the main position was set, an overview stack was recorded at the wavelength 642 nm (SiR-actin; reference run overview) and the focus plane was determined based on this stack. Subsequently, a stack with all four wavelengths was recorded at this point (reference run). About 66s after launch, the in-flight measurements were started and again an overview stack at the wavelength of 642 nm (SiR-actin) was recorded. The vibrations and launch accelerations produced a focus drift, which had to be corrected before the actual measurements. The data were transmitted during the flight to the ground control center at European Space and Sounding Rocket Range (ESRANGE), allowing real-time control, particularly about the focal plane. Thereafter, a total of four measurements were taken at intervals of approximately 50 s at all four wavelengths (t1–4_F). Figure 2 shows the first measurement with all four wavelengths in-flight (t1_F, T+117–142 s after lift-off).

The individual measurements and the corresponding times after lift-off and exposure times to microgravity are listed in Table 3. On the same day of the in-flight measurements, we used three of the ibidi µ-Slides originally prepared as flight reserves for post-flight ground control measurements in the FLUMIAS-engineering model, which is an exact copy of the flight model. Post-flight 1 g measurements were performed to investigate the effect of the measurement conditions (e.g., through bleaching, phototoxicity, cell starving etc.) without the influence of microgravity. Five consecutive measurements according to the flight scenario, but without the overview stacks, were performed for each ibidi µ-Slide. The first measurement was defined as the reference run.

Based on the acquired pictures from the reference runs and the following four acquisitions (in-flight and post-flight on the ground), the volume, surface area and fluorescence intensity of the single cells were determined using the image-analysis software IMARIS. The analysis was performed separately for the nuclear, the lysosomal, the F-actin and the cytoplasm-staining. Based on the cytoplasm-staining, cell heights were determined. Furthermore, an additional analysis was performed for the actin-staining which included the images of the overview-stacks on the ground and in-flight allowing the evaluation of seven time points and total content of polymerized actin (Table 4).

During the analyses of the original unprocessed pre-flight, in-flight and post-flight data we observed a strong bleaching response of all applied live cell dyes in the “fluorescence intensity”. Therefore, we performed a “bleaching correction” calculation for all in-flight and post-flight measurements. The pre-flight reference run (Ref_F) and the first post-flight measurement (Ref_PF) were used as references for bleaching correction of all the in-flight time points and the following post-flight measurements, respectively. In Figure S2 the original unprocessed data and the “bleaching corrected” data of the Calcein cytoplasm staining in-flight and post-flight are demonstrated. The graphs of the fluorescence intensity show that the major part of the bleaching effect could be corrected with the performed calculations.

The “bleaching-corrected” data shown in Figure S4 display the results of individually analyzed human primary macrophage cells. For each cell, the value from the reference run (Ref_F or Ref_PF) was set as 100% and all following data points were referenced to this value, so that relative changes are calculated and differences, due to varying cell sizes are compensated. The comparison of the tested parameters between in-flight and post-flight revealed a clear tendency towards an increase of cell volume and surface area in the cytoplasm staining in microgravity. Until time point t2_F (126–151 s microgravity), the volume and surface area steadily increased with a maximum of 157% and 126% respectively. However, at t3_F and t4_F (201–226 s and 276–301 s microgravity, respectively) the values for cell volume and surface area decreased, reaching the initial values of the 1g reference run. In contrast, in the 1 g post-flight measurements, no increase in cell volume or surface area could be detected. A continuous cell height increase up to 126% could be observed in microgravity, but not in the 1 g post-flight measurements. In microgravity (t1_F and t2_F, 51–76 s and 126–151 s microgravity, respectively), the nuclei volume and surface area tended to increase to maximum values of 150% and 131% respectively, before they returned to the initial values. Again, this effect was not present in the 1 g post-flight measurements. No effect could be detected in the lysosomes and actin (Figure S4).

**Figure 3:**
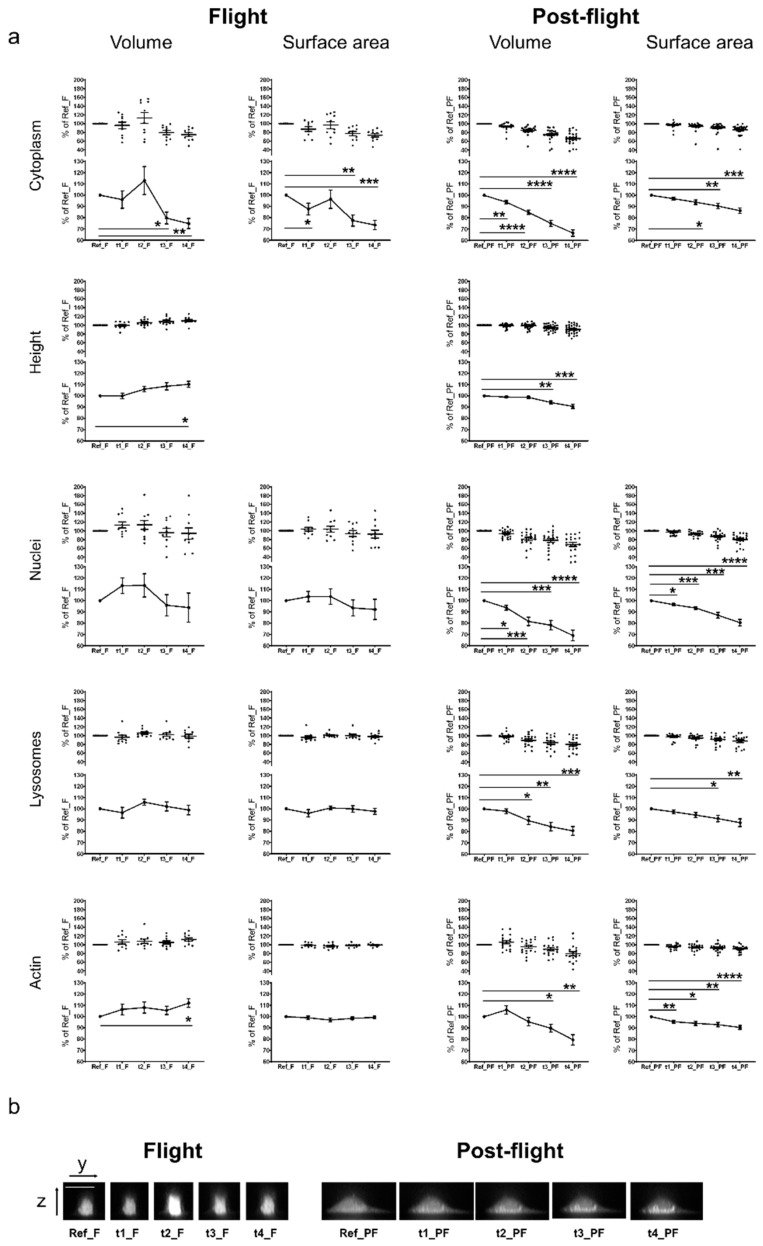
Microgravity-induced changes of cellular and sub-cellular structures. (**a**) Averaged values and statistical evaluation of the single cell analyses. Living human primary macrophages were stained with Nuclear Violet (nuclei staining), Calcein (cytoplasm staining), LysoBrite (lysosome staining), and SiR-actin (F-actin staining) and exposed to microgravity during the TEXUS-54 suborbital ballistic flight. 10 min before the flight and at four times during the flight, confocal microscopic pictures were taken with the confocal laser spinning disk fluorescence microscope FLUMIAS. Additionally, post-flight ground controls were performed. Volume, surface area, and the height of single cells (upper part of each graph) were quantified (software based) after correction of the laser-induced bleaching effect. Averaged values are displayed in the lower parts of the graphs. Error bars represent SEM. *p*-values ≤ 0.05 were considered as significant (*p* ≤ 0.05 = *, ≤0.01 = **, ≤0.001 = ***, ≤0.0001 = ****) (**b**) Sagittal z-stack images of representative example cells stained with the cytoplasmic marker Calcein for all time points of flight and post-flight acquisitions. Scale bar: 20 µm.

In order to quantify the observed cellular effects in microgravity, we averaged the values of the individual data sets and tested for statistical significance using a repeated measurement ANOVA with a subsequent Dunnett’s post-test. Figure 3a shows the graphs of the averaged values of single cell measurements displayed in Figure S4, including the statistical evaluation. The cytoplasmic marker Calcein revealed a significant increase in cell volume with a maximum at t2_F (126–151 s microgravity). At time points t3_F and t4_F (after 201–226 s microgravity), cell volume and surface area significantly decreased below the initial value. In contrast, post-flight 1 g measurements demonstrated a significant signal decrease in all measurement points compared to Ref_PF. The cell height also increased significantly in microgravity from time point t2 (126–151 s microgravity) onwards. In comparison, in the post-flight 1 g measurements, the cell height decreased significantly at t3_PF and t4_PF. An example for representative sagittal z-stack images of cells stained with Calcein for all measurement time points of flight and post-flight acquisitions is shown in Figure 3b. Measurements of stained nuclei in microgravity revealed an increase of the nuclear volume and surface area at t1_F and t2_F (between 51–76 s and 126–151 s microgravity) and a decrease below the initial levels at t3_F and t4_F (after 201–226 s microgravity). The nuclear volume and surface increase were not detectable in the post-flight 1 g measurements, where the measured values for volume and surface area continuously decreased from Ref_PF to t4_PF. No changes were visible in lysosome volume and surface area in microgravity, whereby a significant decrease was detected for time points t2_FP, t3_FP, and t4_FP in the post-flight 1 g measurements. Cell volume, calculated based on actin staining, increased significantly at time point t4_F (276–301 s microgravity). No significant changes were observed for the surface area. Again, a decrease of the actin-based calculated volume and surface area was detected in the post-flight 1 g measurements (Figure 3a).

In contrast to the Nuclear Violet, Calcein and LysoBrite analyses, the SiR-actin analysis additionally contained the reference run overview stack and the flight overview stack, leading to seven measurement points with a labelled actin cytoskeleton for the in-flight sample. As already described, five measurements were performed for the post-flight experiments. All measurements were “bleaching corrected” before the data analysis.

**Figure 4:**
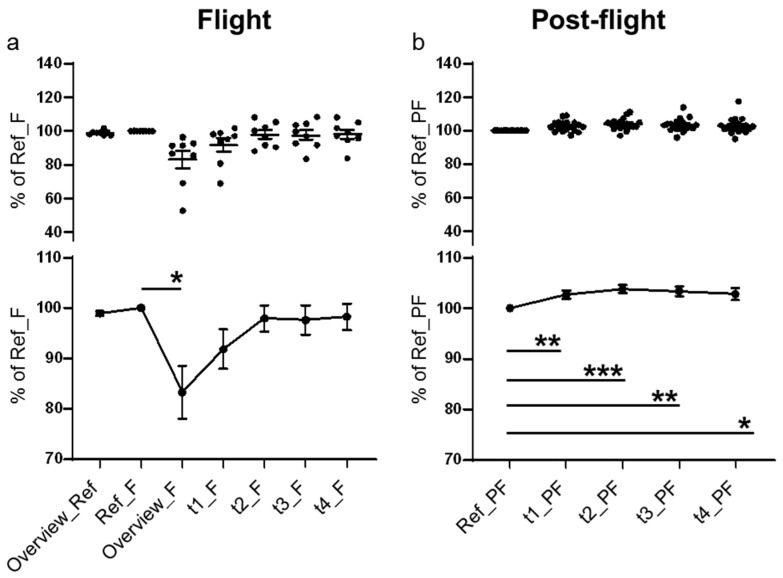
Maximum intensity projection for F-actin. In total seven measurement points were recorded for the actin cytoskeleton labelled with SiR-actin in microgravity and five measurements were performed post-flight. All measurements were “bleaching corrected” before the data analysis. The area covered by the cellular F-actin was determined by comparison of the z-projections of the recorded image stacks for each time point. Upper parts of the graphs show single values and lower parts show averaged values and statistical evaluation of (**a**) the in-flight data and (**b**) the post-flight data. Error bars represent SEM. *p*-values ≤ 0.05 were considered as significant (*p* ≤ 0.05 = *, ≤0.01 = **, ≤0.001 = ***).

Then, a maximum intensity projection (MIP), a volume visualization method for 3D data that projects in a 2D z-projection plane, was performed for each time point. The single cell recordings (Figure S5), as well as the average values demonstrated a clear and significant decrease at the first measurement point in microgravity (Figure 4a), between 4 and 19 s after the onset of microgravity (Table 3; T+70–85 s). At measurement point t1_F (51–76 s after the onset of microgravity), F-actin intensity started to recover and returned to the values of the reference run at time point t2_F (126–151 s after the onset of microgravity) (Figure 4a). In the post-flight recordings, neither the single cell data nor the averaged curve showed this effect (Figure 4b). During the post-flight measurements, the SiR-actin signal constantly increased (Figure 4b).

## 3. Discussion

For live imaging of the cytoskeleton of primary human macrophages, we combined the confocal laser spinning disk fluorescence microscope FLUMIAS with the powerful properties of SiR-actin, a fluorogenic silicon-rhodamine (SiR) conjugated jasplakinolide probe that binds to F-actin and displays minimal toxicity [46], on the TEXUS-54 suborbital ballistic rocket mission. In addition to F-actin, we applied different live-imaging dyes simultaneously for staining of nuclei, cytoplasm and lysosomes and determined the 3D parameter cell volume and cell surface, as well as the cell height in the cytoplasm staining. We detected a tendency towards a short-term increase in cell volume, cell surface and cell height at the 126–151 s microgravity time point (t2_F) in the cytoplasm staining. Thereafter, cell volume and surface area decreased significantly below the initial 1 g values. Cell height increased significantly until the end of the measurements (t4_F; 276–301 s microgravity). The analysis of volume and surface of the nuclei demonstrated a similar effect: A rapid increase after 51–76 s in microgravity (time point t1_F), followed by a decrease below the initial 1 g value at t3 and t4 (after 201 s microgravity). In the visualization of the lysosomes, an increase at 126–151 s microgravity (timepoint t2_F) for volume and surface was only marginally visible. Based on F-actin measurements, the cell volume increased significantly after 126–151 s microgravity (time point t2_F), where no changes were detected regarding the cell surface in-flight.

During post-flight measurements 5 h after the flight, volume and surface area of all investigated structures decreased (Figure 3), probably due to the bleaching of the intracellular dyes (not completely compensated by “bleaching correction”). Without “bleaching correction” all values were higher (Figure S3). Since we performed 3D measurements of living cells in microgravity for the first time, comparisons are only possible with 2D cellular effects in real microgravity of different qualities (10^−2^ to 10^−5^ g), detected after a minimum of 20 s and a maximum of 14 days (Table 5). Rodionova et al. [47] reported an enlarged cellular organelle volume after 14 days of spaceflight, including lysosome like bodies, autophagosomes, vacuoles and vesicles: A reduced cell area was observed after 15 or 30 parabolas (330–660 s of microgravity) [48,49] and after 12 and 24 h spaceflight [50], whereby in our study and cell system, we detected an initial increase and subsequent adaptation, followed by a reduced volume and surface area after 226 s of microgravity. A 30% reduced nuclei size was described after four days and five days of spaceflight [51]. However, in our experiment with primary human macrophages, we detected a rapid initial increase of nucleic volume and surface area, followed by a decrease. In particular, numerous changes of the actin cytoskeleton and microtubules have been described with actin fibers frequently reduced in number and thickness [11,12,51,52,53,54,55,56,57,58,59]. Also “cytoplasmatic holes” have been reported [59,60]. Self-organization of purified tubulin from cow brain was disturbed and impaired [61]. The large variability of morphological and cytoskeletal changes in the microgravity environment in different cell systems suggests cell type-specific responses, which could be explained partially by the different cytoskeletal organization and regulation [15,16,17,18,19,20], actin architecture [21] and microtubule organization [22]. Also, the different physical environments of the different research platforms used (e.g., frequent changes of gravity phase during a parabolic flight, the preceding hypergravity and launch vibration during a suborbital ballistic rocket experiment, or the cosmic radiation during a space flight experiment) may contribute to this heterogenic picture. However, the real-time detection of a rapid cytoskeletal adaptation response in primary human macrophages demonstrated the general biological possibility for rapid cytoskeletal response and adaption reactions to altered gravitational forces. Interestingly, single cell recordings (Figures S2–S5) demonstrated large variability in cell and nuclear morphology and cytoskeletal F-actin constitution. Phenotypic variability in form of spheroid generation has been observed also in MDA-MB-231 [62] and MCF-7 breast cancer cells [63] and FTC-133 thyroid cancer cells [64]. The two morphological phenotypes of MDA-MB-231 breast cancer cells incubated 24h in a random-positioning machine (RPM), are considered as an adaptive, reversible phenomenon of dramatic cytoskeletal reorganization [62]. Phenotypic variability could be explained based on high sensitivity to even small fluctuations of molecular dynamics in response to microenvironmental changes in thermodynamically non-equilibrated and non-linear systems [65].

As demonstrated in Figure 4 (and Figure S5), the actin cytoskeleton responded very rapidly to the microgravity environment with a significant reduction of SiR-actin fluorescence after 19 s microgravity, which subsequently increased again at 51–76 s microgravity (time point t1_F) and returned to initial levels at 126–151 s microgravity (time point t2_F). The SiR-actin probe binds to and stabilizes actin polymers [46,69]. The rapid loss and re-appearance of the SiR-actin fluorescence between 4–19 s and 126–151 s could be therefore the consequence of rapid actin de- and re-polymerization after the onset of microgravity. Rapid responses and adaptation processes between 1 and 42 s were already detected in macrophages previously by our team in the TRIPLE LUX A ISS experiment [25].

Microgravity typically causes cell shape changes and associated cytoskeletal alterations [28,70]. In living cells, the cytoskeletal structure is organized and stabilized with a certain tension level [28,71], facilitating transduction of forces by intermediate filaments and F-actin [71,72]. Force mechanotransduction then results in activation of cytoplasmatic signaling pathways [73,74], cytoskeletal reorganization, remodeling of nuclear morphology, intermingling of chromosome territories [75] and chromatin condensation [76], accompanied by differential gene expression patterns [77], as recently detected in cells of the monocyte-macrophages system [26].

In our study, we found alterations in cell geometry and rapid cytoskeletal re-organization in primary human macrophages in real microgravity by real time imaging experiments in space, supporting previous findings that gravity plays a fundamental role in shaping form and function in living systems [62]. Whereby the results of our ISS experiment CELLBOX-PRIME [23] demonstrated a structurally intact actin and vimentin cytoskeleton after 11 days in microgravity, we detected a rapid adaptation response of the actin cytoskeleton in less than 3 min microgravity in the FLUMIAS TEXUS-54 suborbital rocket experiment. While the adaptive response of the human physiological systems in microgravity requires hours until weeks [78], only a very few studies addressed cellular adaptation [23,25,26,79]. In the current study, we found that the actin cytoskeleton, representing the potential gravity-sensitive cellular structure [28,29] adapted very rapidly to the new microgravity environment in primary human macrophages, reaching probably a stable steady state [23].

## 4. Materials and Methods

### 4.1. FLUMIAS Confocal Laser Spinning Disk Fluorescence Microscope

The FLUMIAS microscope was designed and built by Airbus DS (Bremen, Germany). As a principal item for FLUMIAS microscope, the iMIC microscope developed and built by the company FEI Munich GmbH (Munich, Germany; now part of Thermo Fisher Scientific) was used and certain components were re-designed and adjusted for the application on a ballistic rocket. In total, two versions of the FLUMIAS microscope were built, a flight model (FM) and an engineering model (EM), the latter can also be used on parabolic flights. As a confocal spinning disk microscope, FLUMIAS enables the parallel scan of 1200 sample points per field of view leading to a fast image generation. The sample holder allowed the integration of a standard ibidi ibiTreat µ-Slide 0.4 (ibidi µ-Slide) (ibidi, Martinsried, Germany), which contains six channel-shaped cell culture cavities (ibidi channels) that can be loaded. The x-y stage provided a total travel distance of approximately 24 mm in both directions enabling the measurement of three out of the six ibidi channels of the ibidi µ-Slide. Guiding elements guaranteed position stability of 10µm in the x-y direction. The FLUMIAS microscope comprised a Carl Zeiss water immersion 40×/1.2 W Corr objective mounted on a voice coil focus drive with an optical sensor with a resolution of 20nm that allowed movements in the z-direction and prevented potential image scaling errors, due to environmental influences like altered gravity or vibrations. Four different light sources were implemented: Three diode lasers for 405 nm (120 mW), 488 nm (200 mW), 642 nm (140 mW) and one diode-pumped solid-state laser for 561 nm (150 mW). Furthermore, Airbus DS included an experiment service subsystem that provided e.g., an experiment timer, a water-cooling circuit, a computer with the control software for the microscope, and storage capacity for high resolution images. Before the first application of the FLUMIAS-TEXUS microscope a system calibration called “end of line test” was performed by FEI, Thermo Fisher Scientific. This test included quantitative image correction and calibration for confocal fluorescence microscopy using thin reference layers and Sectioned Imaging Property (SIP) chart-based calibration procedures [80].

A detailed description of the FLUMIAS microscope developed and built by Airbus DS (Bremen, Germany) can be found in Reference [11].

### 4.2. Isolation of Monocytes

Human monocytes were isolated from anonymized buffy coats, a by-product of blood donations, received from the blood transfusion service (Zurich, Switzerland; internal project registration no. 579) following a standardized protocol. Buffy coats were diluted 1:2 with sterile 1× PBS (Biochrom GmbH, Berlin, Germany). 15 mL of this solution was layered carefully on 10 mL Ficoll Paque Premium (GE Healthcare Bio-Sciences, Uppsala, Sweden) and centrifuged at 400× *g* for 30 min at RT without break. The PBMC layer visible at the interphase was collected in a new sterile tube, sterile PBS was added up to a total volume of 50 mL followed by a second centrifugation step at 400× *g* for 10 min at RT. The supernatant was removed, the cell pellet was resuspended in 5 mL PBS (Biochrom GmbH, Berlin, Germany) and 45 mL 1× PBS was added before centrifugation at 350× *g* for 10min at RT. This washing step was repeated twice. The cell pellet was resuspended in 20 mL Mononuclear cell medium (Promocell, Heidelberg, Germany) and carefully layered on 25 mL of a 46% Percoll solution (10.64 mL Percoll (GE Healthcare Bio-Sciences, Uppsala, Sweden), 0.86 mL 10× PBS (Sigma-Aldrich Chemie GmbH, Steinheim, Germany), 13.5 mL RPMI (Biochrom GmbH, Berlin, Germany)). Samples were centrifuged at 550× *g* for 30 min at RT without break. Monocytes concentrated at the interphase were collected, transferred to a new sterile tube and 1× PBS was added up to a total volume of 50 mL. The cell suspension was centrifuged at 400× *g* for 10 min at RT. In case the supernatant was still turbid the washing step was repeated. The cell pellet was resuspended in 8 mL MCM (Promocell, Heidelberg, Germany), viable cells were counted in a Neubauer chamber. Aliquots of 22.5 million cells were prepared, centrifuged at 350× *g* for 10 min at RT, cell pellets were resuspended in 1.5 mL Cryo Serum Free Medium (Promocell, Heidelberg, Germany) and frozen at −80 °C. One day after freezing vials was transferred to −150 °C for storage. Cells were transported frozen and temperature controlled (−80 °C) from the home laboratory to the ESRANGE Space Center laboratories in Sweden.

### 4.3. Differentiation of Primary Human Macrophages

In the laboratories of the ESRANGE Space Center (Sweden) primary human M1 macrophages were differentiated from frozen monocytes according to the standard protocol provided by Promocell (Heidelberg, Germany). Briefly, cryovials with frozen monocytes (volume 1.5 mL) were thawed in a water bath at 37 °C for 2 min with gentle agitation shaking. The cell suspension was immediately transferred into 20mL of MCM (PromoCell, Heidelberg, Germany) pre-equilibrated for at least 20 min in a 37 °C CO_2_ incubator. After 8–16 h incubation (37 °C, 5% CO_2_, 95% humidity), cells in MCM were collected in a sterile tube and centrifuged at 350× *g* for 10 min at RT. The cell pellet was resuspended in 8 mL MCM (Promocell, Heidelberg, Germany) and viable cells were counted in a Neubauer chamber. The cell concentration was adjusted with M1 Macrophage Generation Medium DXF to 1.2 million cells per mL. Three milliliters (3.6 million cells) of this suspension was transferred into each well of an 8 well plate (Thermo Fisher Scientific, Rochester, New York, NY, USA). Alternatively, 20,000 to 35,000 cells were seeded into the channels of an ibidi µ-Slide and incubated for six days (37 °C, 5% CO_2_, 95% humidity). On day 6, a partial medium exchange was performed by replacing 50% of the M1 Macrophage Generation Medium DXF in each well. On day 9, a complete medium exchange was performed. Therefore, the medium from each well, including cells in suspension, was collected in a sterile tube. Immediately, 0.5 mL of fresh M1 Macrophage Generation Medium DXF was added to the adherent cells in the 8 well plate. The collected cells in suspension were centrifuged at 350× *g* for 15 min at RT. The cell pellet was resuspended in 3 mL fresh M1 Macrophage Generation Medium DXF and transferred back to the original well. From day 10 on, macrophages were ready-to-use for the experiments.

### 4.4. Experiment Preparation Protocol and Mission Scenario

Primary human macrophages were seeded in ibidi µ-Slides either at differentiation day 0 or at day 9 during the complete medium exchange (see Section 4.3). In the latter case residual adherent cells were detached by rinsing the bottom of the 8 well plate. 20,000 to 35,000 cells were plated per ibidi channel and incubated at 37 °C in a CO_2_ incubator in a humid chamber. Cells were visually inspected and evaluated under a light microscope on a daily basis and the medium was exchanged as required. For the TEXUS-54 suborbital rocket flight experiment six ibidi µ-slides with differentiated human primary M1 macrophages (>10 days) were selected and stained 24 h before lift-off with 100 nM SiR-actin (Spirochrome, Stein am Rhein, Switzerland) in M1 Macrophage Generation Medium DXF and stored in a CO_2_ incubator at 37 °C. We applied the dye at very low concentrations (100 nM) in order to not affect the F-actin dynamics [69]. At T −5.5 h before launch, cells in the different ibidi µ-slides were evaluated under the light microscope, and the medium of the selected ibidi µ-Slide was exchanged for M1 Macrophage Generation Medium DXF containing 100 nM SiR-actin (Spirochrome, Stein am Rhein, Switzerland), 0.5× LysoBrite Orange (stock concentration 500×; AAT Bioquest, Sunnyvale, California), 5 µM Nuclear Violet LCS1 (AAT Bioquest, Sunnyvale, California), and 1 µM Calcein AM (Thermo Fisher Scientific, Allschwil, Switzerland) and 30 mM HEPES (L-1613, GmbH, Berlin, Germany). The two outlets of the ibidi channels were closed by luer connectors (10824, ibidi, Martinsried, Germany) and medium filled connecting tubes of 0.8mm diameter (10841, ibidi, Martinsried, Germany) (Figure 1). The ibidi µ-Slide was mounted into the late access unit and the cells were evaluated in the FLUMIAS microscope engineering model. At T −1.5 h the ibidi µ-Slide in the late access unit was integrated via the late access port into the pre-warmed (36.5 °C) FLUMIAS flight model in the launch tower. At T −1 h three different areas/positions on the sample with representative cells were selected, one primary position and two reserve positions. At T −10 min a stack of microscopic pictures was acquired using the 642 nm wavelength laser that illuminates the SiR-actin staining. This stack spans 90 µm with 91 pictures and is termed “reference run overview stack”. Based on this stack and using a custom-made software program, a focus plane was chosen for the subsequent acquisition of a stack of pictures in all four wavelengths (405, 488, 561, and 642 nm), spanning 25.28 µm in 80 pictures, enabling a high resolution of cellular structures also in the z direction. This stack is termed the “reference run”. After lift-off and the initial hypergravity phase, the in-flight measurements were started shortly after entering the microgravity phase. First, an overview stack was recorded using the 642 nm wavelength laser that illuminates the SiR-actin staining (spanning 90 µm in 91 pictures analogous to the reference run overview stack) termed “Flight overview” stack. Pictures of this stack were transmitted live to the ground control center at ESRANGE by telemetry, enabling adjustments of the focal plane, which could shift due to the force conditions of the launch and flight of the ballistic rocket. Four consecutive measurements were performed with all four wavelengths (405, 488, 561, 642 nm) spanning 25.28 µm in 80 pictures termed flight measurement time points t1_F–t4_F. The individual measurements were separated by 50 s, due to rotating measurement times between the three science teams. Values for laser intensities and exposure times, as well as information on z-step size can be found in Table 2. The remaining seeded ibidi µ-slides not used for the flight experiment were used for post-flight ground control measurements.

### 4.5. TEXUS-54 Suborbital Ballistic Rocket Mission Profile

TEXUS-54 was a German Center for Aerospace (DLR) funded suborbital ballistic rocket mission. The vehicle assembly comprised the experiment payload on top of a two-stage VSB-30 rocket motor. The rocket launch was performed on 13 May 2018 at 10:30 from ESRANGE Space Center in Sweden. Excerpt of the flight profile: (i) Altitude: 240 km; (ii) total microgravity time: 353 s (10^−5^ g); (iii) 8.4 g first-stage peak thrust acceleration; (iv) 5.4 g mean thrust acceleration; (v) first-stage burnout at 11.9 s, (vi) first stage separation at 12.1 s; (vii) 12.0 g second-stage peak thrust acceleration; (viii) 6.6 g mean thrust acceleration; (ix) burnout at 42.9 s; (x) yo-yo despin at 56.0 s; and (xi) second stage separation at 59.0 s.

### 4.6. Post-Flight Measurements

Three ibidi µ-slides that were prepared as flight reserves were used for post-flight measurements on the day of the TEXUS-54 launch. Five consecutive measurements analogous to the flight scenario but without overview stacks were performed per ibidi µ-slide in the FLUMIAS EM. The first measurement was defined as the reference run (Ref_PF).

### 4.7. Quantitative Analysis of Microscopic Images

The images recorded with the FLUMIAS microscope were analyzed with the image processing software Imaris 9.2.0 (Bitplane). Only cells which were depicted as a whole in the 3-D reconstruction were analyzed, cells which were depicted incompletely were excluded. For all four stained epitopes (nuclei, cytoplasm, actin and lysosomes) the volume, surface area, and mean fluorescence intensity (MFI) were quantified for single cells as follows: For each 3-D picture, a template for the surface was created with the surface function. Smoothing of 0.5 was chosen and the coverage of mask and cells was adjusted manually. To discriminate single cells the function “split touching objects” was enabled and seed points of 15–20 µm were chosen. The filter “quality above threshold” was adjusted so that the single cells were recognized. False positive or unstained cells and unspecific labels were excluded manually. Values of volume, surface area, and MFI were exported.

Additionally, the cell height was determined: Based on the cytoplasm staining in the “Slice view”-function, polygons were set at the lower and upper borders of the single cells and the distance was measured.

On the pictures of the in-flight sample, the field of vision contained twelve complete cells. Depending on the stained structure, 8–10 cells could be analyzed in all measurements. From the post-flight measurements, 18–29 cells could be analyzed, including all three measurement runs. In all measurements in-flight and post-flight on the ground we observed photo bleaching between two consecutive measurements for all dyes. Therefore, we performed a “bleaching correction” for all measurements. Analyses were performed for both data sets, the uncorrected and the “bleaching corrected” data. Cell volume, surface area and fluorescence intensity were measured for all 3D image series. In the case of the SiR-actin staining, an additional analysis was performed with ImageJ. MIP (maximum intensity projection) images were generated with the z-projection program tool. The ROI (region of interest) manager was subsequently used to measure the area of each cell for each time point.

### 4.8. Bleaching Correction

The stacks were corrected for intensity changes with ImageJ. Photo bleaching is usually the main reason for decreasing fluorescence signals. In a first step each channel of each z-stack is reduced to a mean z-projection image. In case the stack has a larger z-range and spacing the mean image is created from a similar sub stack range. In the mean projection image, a region with a background signal and a region with a homogenous bright signal was selected. The complete time series of one color was used for the selection of these two regions to avoid the influence of drift and shape changes. Mean values for signal S(c,t) and background B(c,t) were determined from the selected regions, where c= channel and t = time.

Linear scaling factors f(c,t) and offsets o(c,t) were determined for all colors and time points to match the signal and background of the reference run. The reference run is defined as the first stack with all four colors.

S(c,t) = S(c,tref)*f(c,t) + o(c,t)

B(c,t) = B(c,tref)*f(c,t) + o(c,t)

Finally, the corrections are applied to the stack data by multiplication with f and adding the offset o.

Icorr(c,t) = I(c,t)*f(c,t) + o(c,t)

### 4.9. Statistical Analysis

Statistical evaluation was performed with the software GraphPad Prism 5.0 (GraphPad software Inc., San Diego, CA, USA). To compare cell volume, surface area, and cell height in Figure 3, a repeated measurement ANOVA with a subsequent Dunnett’s post-test was used. A paired t-test was applied to compare the cell areas identified by the maximum intensity projection (Figure 4). Each time point was compared to the respective reference run value of the flight and post-flight samples. P-values ≤0.05 were considered as significant (≤0.05 = *, ≤0.01 = **, ≤0.001 = ***, ≤0.0001 = ****).

## Figures and Tables

**Table 1:** Overview of applied live cell staining dyes, concentrations and staining times.

Staining Dye	Cellular Organelle	Final Concentration	Staining Time before Lift-Off
SiR-actin	F-actin	100 nM	24 h
Calcein	Cytoplasm	1 µM	5.5 h
Nuclear Violet	Nuclei	5 µM	5.5 h
LysoBrite	Lysosomes	0.5×	5.5 h

**Table 2:** Image acquisition parameters with the confocal laser spinning disk fluorescence microscope FLUMIAS on the TEXUS-54 suborbital rocket flight mission.

Parameter	Value	Comment
Excitation wavelength [nm]	405/488/561/642	Cell organelle/structure visualized: nucleus/cytoplasm/lysosomes/F-actin
Exposure time [ms]	15/15/50/50	-
Laser intensity [%]	100/100/20/100	-
X/Y-step size [µm]	10	-
Z-step size [µm]	1	-
1. Z-stack: Z-stack height [µm]	90	Reference run overview stack/in-flight overview stack
1. Z-stack: Image-to-image distance [µm]	1	-
Image-# 1. Z-stack	91	-
Z-stack height [µm]	25.28	-
Image-to-image distance height [µm]	0.32	-
Image number per Z-stack	80	-
Number of Z-Stacks per acquisition	1	In total four acquisitions (t1–t4) with four wavelengths in-flight
Image-# per loop	320	four wavelengths each 80 images
Image-# per run	1280	four loops (t1–t4), each loop with four wavelengths each with 80 images
Acquisition time [s]	21.58	-
Working mode (nominal)	Z-Stack	-

**Table 3:** Microscopic image stack acquisition during the FLUMIAS TEXUS-54 experiment. Microgravity exposure times are calculated from the start of the microgravity phase until the end of the respective measurement.

Flight Phase	Image Stack Acquisition (Used Wavelength)	Time with Respect to Lift-Off	Comment
Pre-flight	Reference run overview stack (642 nm)	T-10 min	Pre-flight 1 g ground control
Pre-flight	Reference run (Ref_F) (405, 488, 561, 642 nm)	T-9 min	Pre-flight 1 g ground control
Lift off	-	T0	-
Onset of microgravity	-	T+66 s	-
In-flight	Flight overview stack (642 nm)	T+70–85 s	4–19 s microgravity
In-flight	Flight measurement t1 (t1_F) (405, 488, 561, 642 nm)	T+117–142 s	51–76 s microgravity
In-flight	Flight measurement t2 (t2_F) (405, 488, 561, 642 nm)	T+192–217s	126–151 s microgravity
In-flight	Flight measurement t3 (t3_F) (405, 488, 561, 642 nm)	T+267–292s	201–226 s microgravity
In-flight	Flight measurement t4 (t4_F) (405, 488, 561, 642 nm)	T+342–367 s	276–301 s microgravity
Post-flight	3 Post-flight (PF) runs with five measurements each: Ref_PF, t1_PF, t2_PF t3_PF, t4_PF (405, 488, 561, 642 nm)	T+5 h–5 h 45 min	Post-flight 1 g ground control

**Table 4:** Overview of analyzed parameters and number of measurements/time points of the investigated cellular components.

Cellular Component	Volume	Surface Area	MIP	Height	Measurements/Time Points
Nuclei	x	x	-	-	5
Cytoplasm	x	x	-	x	5
Lysosomes	x	x	-	-	5
F-actin	x	x	x	-	5/7 *

* Including reference run overview stack & in-flight overview stack; MIP = maximum intensity projection.

**Table 5:** Literature overview of the effects of microgravity on cell organelles, structures and morphology observed in mammalian cells or molecules exposed to real microgravity on different platforms (PF: Parabolic flight, SR: Suborbital rocket, SF: Space flight).

Effects on Cells, Cell-Organelles, and Cell-Structures	Cell Type	References	Microgravity Platform	Microgravity Exposure Time
**Cell morphology**				
Cytoplasmic retraction and membrane ruffling, decreased cell area	Osteosarcoma cells (ROS 17/2.8)	[48]	PF	15 parabolas, 30 parabolas
Decreased cell area	Osteosarcoma cells (ROS 17/2.8)	[49]	PF	15 parabolas, 30 parabolas
No shape change at two days microgravity	Osteosarcoma cells (ROS 17/2.8)	[66]	SF	two days
Shape change at 4 and 6 days microgravity: Round, increase in microvilli, three sub-groups of morphology (1) long cytoplasmic extensions; (2) round piling cells, unable to flatten; (3) normal spread out cells, resembling the ground controls	Osteosarcoma cells (ROS 17/2.8)	[66]	SF	four days, six days
Decrease of cell area, number of vinculin spots and mean vinculin spot area	Osteosarcoma cells (ROS 17/2.8)	[57]	SF	12 h, 24 h
Decreased cell area, decreased number of vinculin spots per cell, decreased mean vinculin spot area, actin and focal adhesion decreased, fewer stress fibers, vimentin and microtubule network no major differences (12 h and 24 h)	Osteosarcoma cells (ROS 17/2.8)	[50]	SF	12 h, 24 h
Contracted, roundish cell shape with short protrusions	J-111 cell line	[52]	SF	one day
**Nuclear shape**				
Reduced nuclei size by 30%, oblong shape, less punctate areas, actin cytoskeleton with a reduced number of stress fibers	MC3T3-E1 osteoblasts	[51]	SF	four days
High variability, many smaller and condensed and some fragmented nuclei, larger intact nuclei with larger diameter	Primary mouse osteoblasts, RAW 264.7 murine macrophage cell line	[58]	SF	five days
**Other cellular organelles**				
Increased cellular organelle volume of lysosome-like bodies, autophagosomes, Golgi complex, vacuoles and vesicles	Osteocytes, ileac crest of monkeys	[47]	SF	14 days
**Cytoskeleton**				
Actin cytoskeleton with a reduced number of stress fibers perinuclear actin localization, lamellipodia	MC3T3-E1 osteoblasts	[51]	SF	four days
No changes in actin structure	Human Jurkat T cells	[10]	SR	12 min
Localization of actin at cell border, contracted cell shape, changed the distribution of F-actin and tubulin filaments, no strong bundles, fewer lamellipodia	J-111 cell line	[52]	SF	one day
Accumulation of F-actin at the cell membrane, increase in F-actin around nucleus	Human endothelial cells	[53]	PF	one parabola
Rearrangement of the actin network, perinuclear clustering	ML-1 follicular thyroid cancer cells	[54]	PF	one parabola
Disturbance of actin bundles and cytoplasm discontinuity, disappearance of the microvilli or filopodia- and lamellipodia-like structures	follicular thyroid cancer cells FTC-133	[11]	PF	1–2 parabolas
Disturbance of actin bundles, formation of filopodia- and lamellipodia like structures, cellular detachment	follicular thyroid cancer cells FTC-133	[11]	SR	six min
No changes of the actin and vimentin cytoskeleton structure	Primary human macrophages	[23]	SF	11 days
Formation of thick vimentin and tubulin bundles, formation of aggregates of proteins, due to de-polymerization and discontinuities of the filamentous network	Human T lymphocytes from blood donations	[67]	SR	30 s
Disorganization of microtubules	Human Jurkat T cells	[12]	SF	four h
Vimentin structural changes, increased appearance of large bundles	Human Jurkat T cells	[10]	SR	12 min
No microtubule self-organization and local disorder	Purified tubulin from cow brains	[61]	SR	13 min
Altered microtubule cytoskeleton after 4 h and partial re-organization after 48 h	Human Jurkat T cells	[55]	SF	four h, 48 h
Disoriented microtubule	Breast cancer MCF-7	[56]	SF	1.5 h
Presence of thicker, bundled microtubule, smaller focal adhesion size, reduced cortical actin, fewer actin stress fibers with reduced fiber thickness, a significant increase in cell area measured with phalloidin	Primary mouse osteoblasts, RAW 264.7 murine macrophage cell line	[58]	SF	five days
Disorganized sarcomeric structure with interfilament holes	C57BL/6N mice Cardiac muscle	[60]	SF	30 days
Cell cytoplasm discontinuity, holes in the microtubule network, absence of stress fibers, actin network rearrangement and ring formation around the cell membrane	Human chondrocytes	[59]	PF	one parabola, 31 parabolas
No changes of the actin cytoskeleton structure	Primary humane fibroblasts	[68]	SF	three days, 14 days

## Supplementary Materials

Supplementary materials can be found at https://www.mdpi.com/1422-0067/20/10/2402/s1.

## Abbreviations

Airbus DS	Airbus Defense and Space
FLUMIAS	Fluorescence-Microscopic Analyses System for Life-Cell-Imaging in Space
TEXUS	German: Technologische Experimente unter Schwerelosigkeit
DLR	German Aerospace Center
ESRANGE	European Space and Sounding Rocket Range
MFI	Mean Fluorescence Intensity
MIP	Maximum Intensity Projection
MORABA	DLR Mobile Rocket Base
ROI	Region of Interest
SEM	Standard Error of the Mean
SSC	Swedish Space Cooperation
