# Supplementary material for: Rapid Morphological and Cytoskeletal Response to Microgravity in Human Primary Macrophages
Source: Int J Mol Sci. 2019 May 15;20(10):2402. doi: 10.3390/ijms20102402 (PMC6567851; doi:10.3390/ijms20102402)
Supplement: Supplementary file 1 [file ijms-20-02402-s001.pdf]

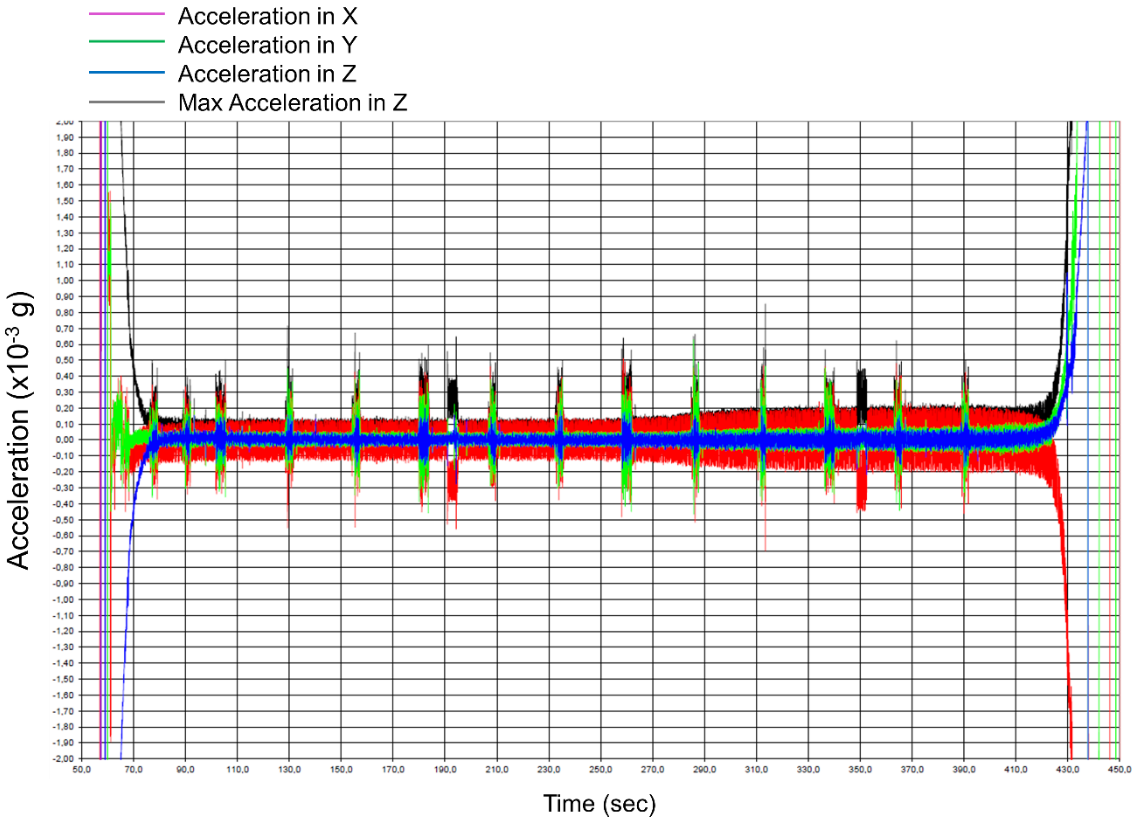


**Figure S1**. Gravity profile of the TEXUS-54 flight. Recording provided by OHB System AG.

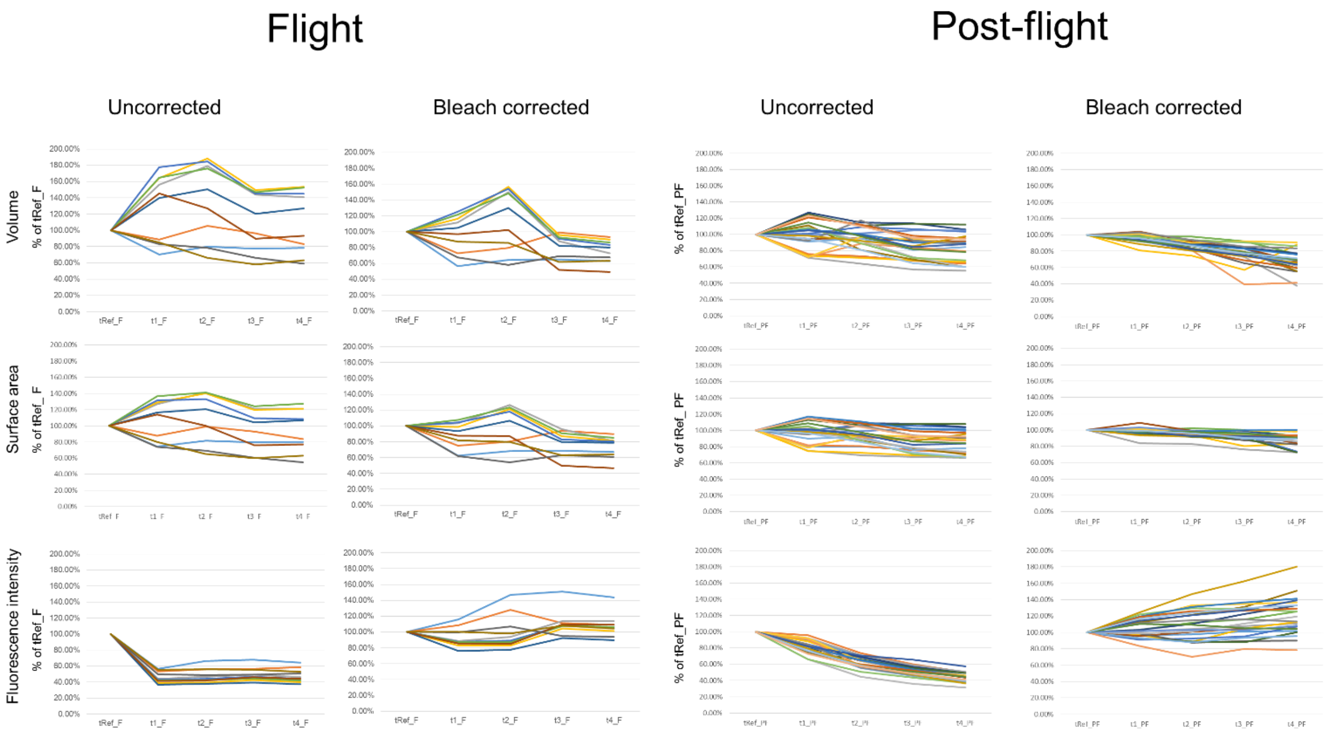


**Figure S2**. Human primary macrophages were fluorescently stained with Calcein (cytoplasm staining) and exposed to microgravity during a suborbital ballistic rocket flight. 10min before the flight and at 4 times during the flight confocal microscopic pictures were taken with the FLUMIAS microscope. Additionally, post flight ground controls were performed. Using image processing software volume, surface area and fluorescence intensity of single cells were quantified from the original pictures and after correction of the laser-induced bleaching effect that occurs when taking pictures of the same area several times. The displayed graphs allow the comparison of the cell parameters in flight and post flight for the original and bleaching corrected data.

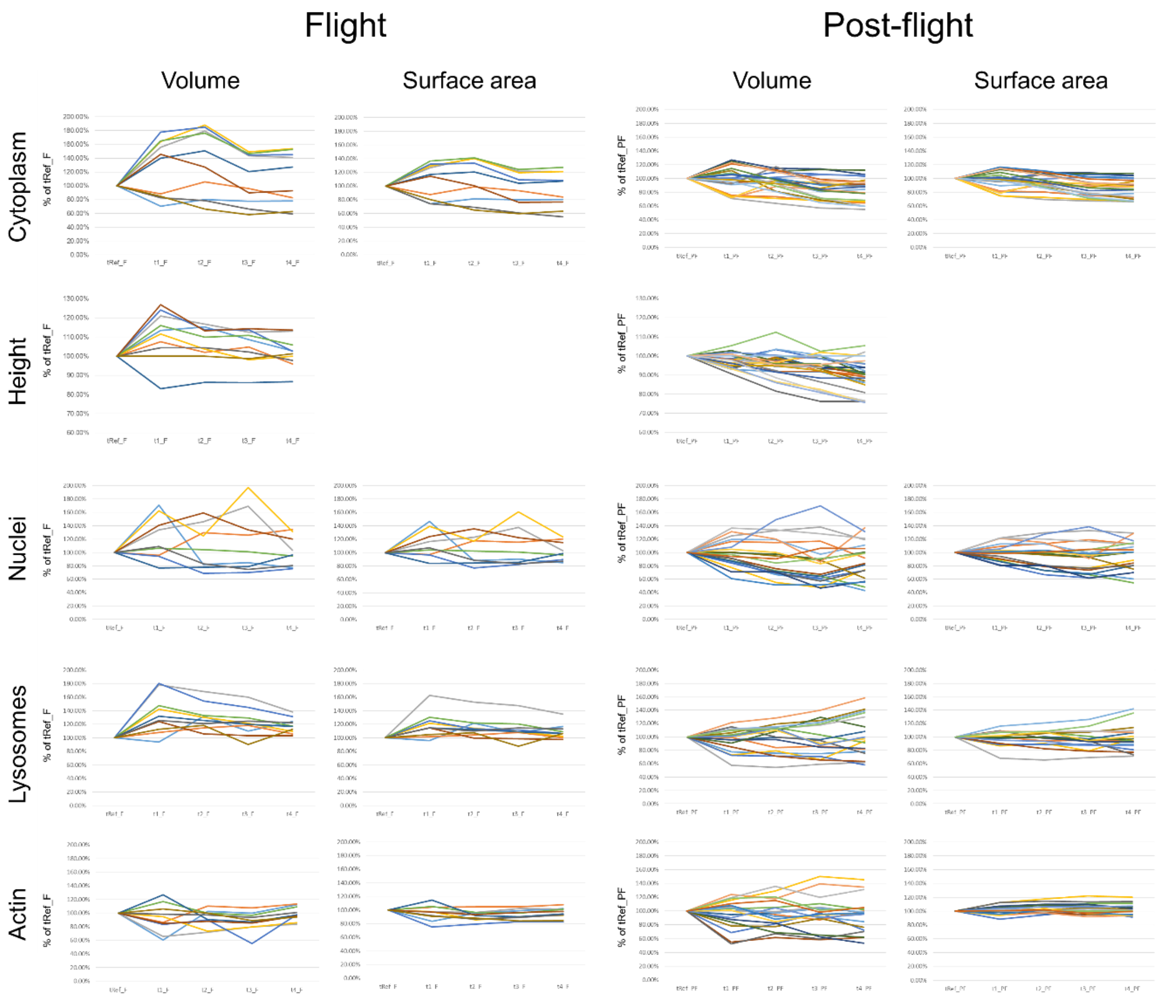


**Figure S3.** Human primary macrophages were fluorescently stained with Nuclear Violet (nuclei staining), Calcein (cytoplasm staining), LysoBrite (lysosome staining), and SiR-actin (F-actin staining) and exposed to microgravity during a suborbital ballistic rocket flight. 10min before the flight and at 4 times during the flight confocal microscopic pictures were taken with the FLUMIAS microscope. Additionally, post flight ground controls were performed. Volume, surface area, fluorescence intensity and the height of single cells were quantified software based on the original non-bleaching corrected data.

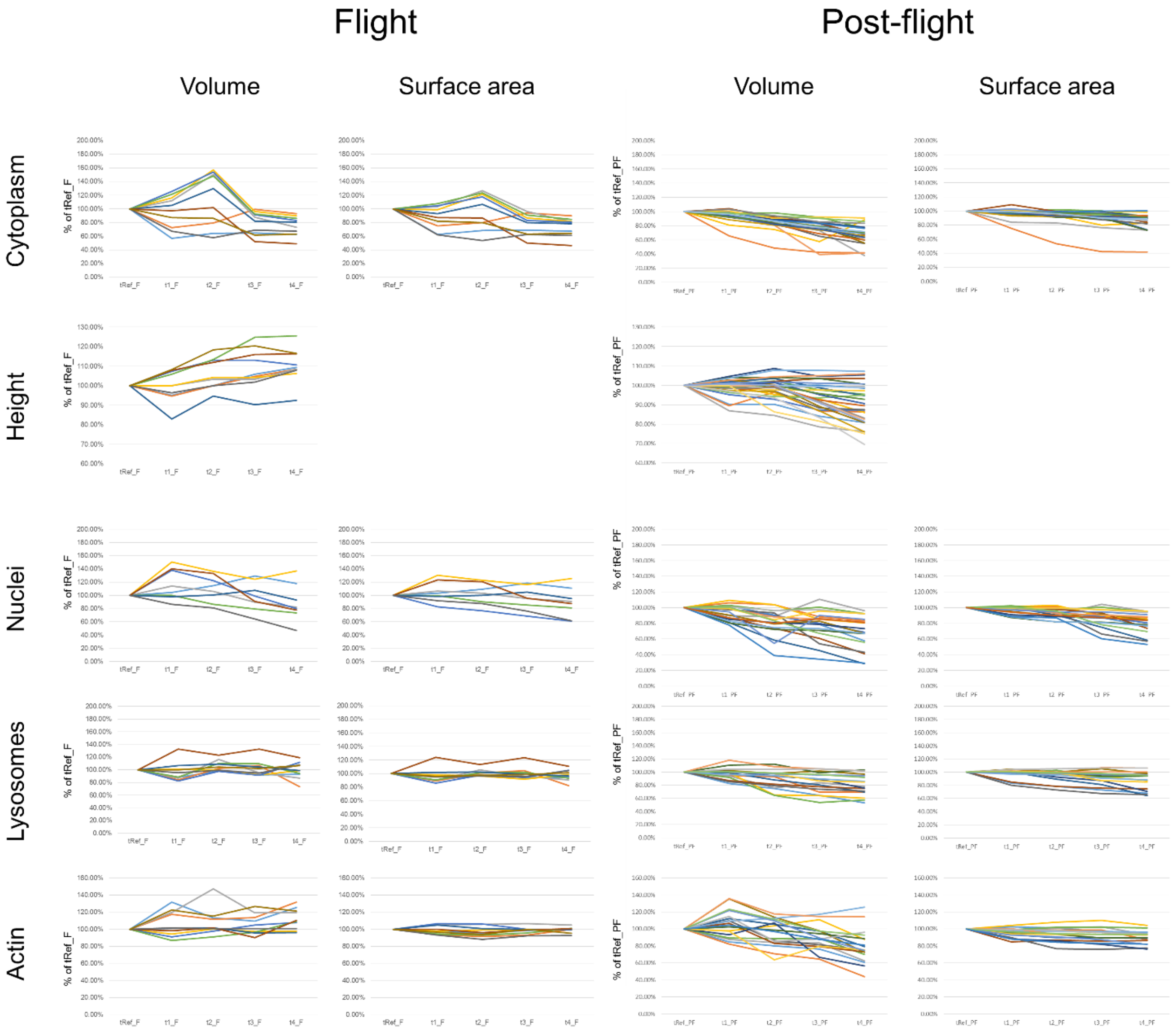


**Figure S4**. Human primary macrophages were fluorescently stained with Nuclear Violet (nuclei staining), Calcein (cytoplasm staining), LysoBrite (lysosome staining), and SiR-actin (F-actin staining) and exposed to microgravity during a suborbital ballistic rocket flight. 10min before the flight and at 4 times during the flight confocal microscopic pictures were taken with the FLUMIAS microscope. Additionally, post flight ground controls were performed. Volume, surface area, fluorescence intensity and the height of single cells were quantified software based after correction of the laser-induced bleaching effect.

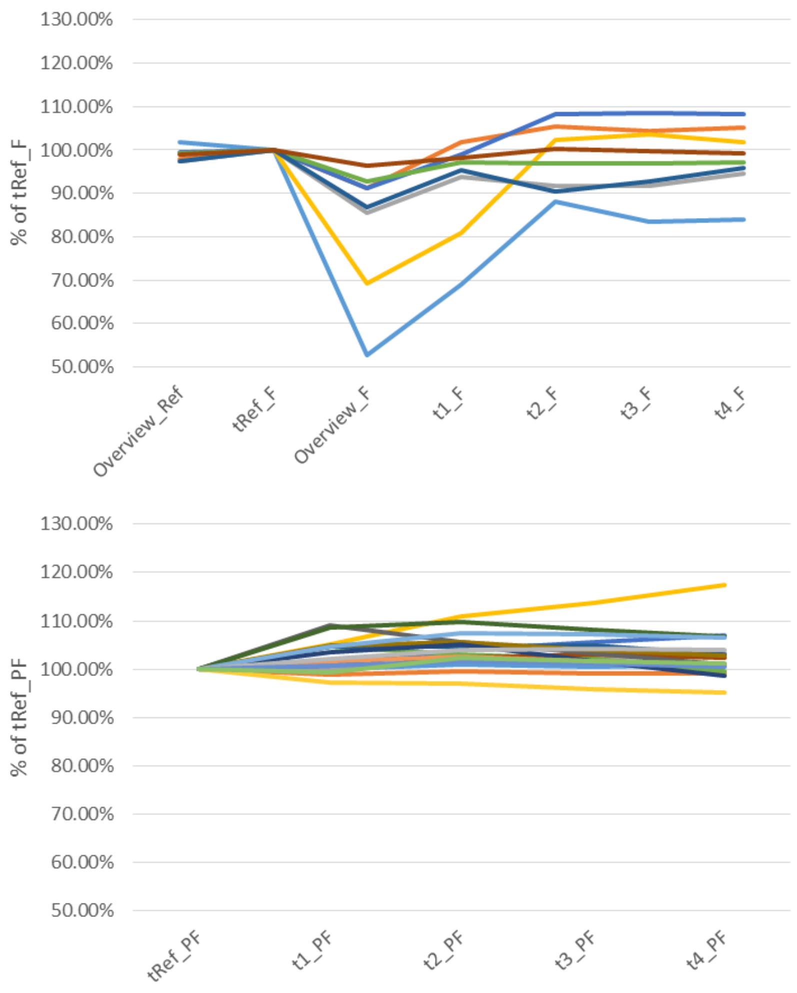


**Figure S5**. In total seven measurement points were recorded for the actin cytoskeleton labelled with SiR-actin in microgravity and five measurements were performed post flight. All measurements were bleaching corrected before data analysis. The area covered by the cellular F-actin was determined for each time point by determining the z-projections of the recorded image stacks. Single cell recordings of the flight sample (top) and the post flight samples (bottom).

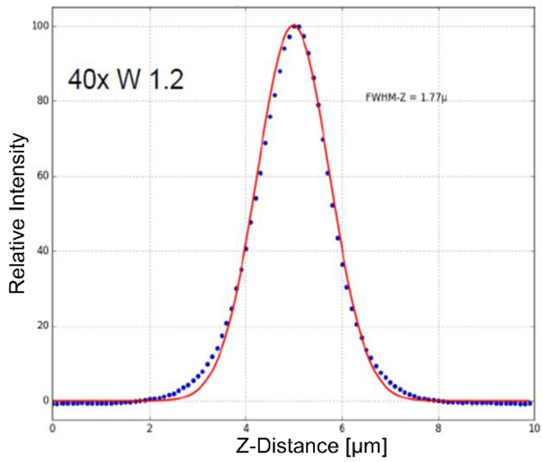


FWHM=1.77 µm

**Figure S6.** A typical confocality calibration curve of the FLUMIAS-TEXUS microscope system. The fluorescence signal of a thin layer is plotted as a function of the objective lens' z-position. The fluorescing thin layer was excited with 488nm and the signal was recorded with a sCMOS camera. The blue dots represent single measurement points with a 100nm step size. The red curve represents the mathematical interpolation.
